# d-lactate drives lysine d-lactylation to regulate metabolism in *Escherichia coli*

**DOI:** 10.1016/j.jbc.2026.111374

**Published:** 2026-03-16

**Authors:** Jianji Zhang, Yong Zang, Zhiqing Yu, Fei Zhao, Jingya Wu, Yuhan Wang, Aiyuan Wang, Guijin Zhai, Yanpu Han, Chen Chen, Jinjun Ye, Hanyang Dong, Kai Zhang

**Affiliations:** 1 Key Laboratory of Immune Microenvironment and Disease (Ministry of Education), Tianjin Institute of Immunology, The Province and Ministry Co-sponsored Collaborative Innovation Center for Medical Epigenetics, School of Basic Medical Sciences, Tianjin Medical University, Tianjin, China; 2 Department of General Surgery, Longgang Central Hospital of Shenzhen, Shenzhen, China; 3 Key Laboratory of Breast Cancer Prevention and Therapy (Ministry of Education), Tianjin Medical University Cancer Institute and Hospital, Tianjin Medical University, Tianjin, China

**Keywords:** post-translational modifications, lysine d-lactylation, bacteria, metabolism, proteomics

## Abstract

l-lactate-derived lysine l-lactylation (K_L-la_) has emerged as a key regulator in diverse cellular processes and disease pathogenesis. While lactate predominantly exists as the l-isomer in eukaryotes, both l- and d-lactate are present in some bacteria. However, it remains unclear whether d-lactate can drive post-translational modification to exert biological functions. Here, we reported that d-lactate-derived lysine d-lactylation (K_D-la_) serves as a post-translational modification in *Escherichia coli*. We demonstrated that acetate CoA-transferase (YdiF) catalyzes the formation of d-lactyl-CoA, the key d-lactyl donor, connecting d-lactate to K_D-la_. Notably, we identified 86 K_D-la_ sites on 71 proteins in *E. coli*. In addition, our data demonstrated that anerobic conditions enhance glycolysis, increasing d-lactate production *via*l-lactate dehydrogenase A (LdhA) and further elevating K_D-la_ levels. We also found that CobB functions as an endogenous de-d-lactylase. Our experiment further showed that K257 of GapA can regulate bacterial growth, whereas CobB can remove K_D-la_ at this site, suggesting a potential CobB-mediated K_D-la_ role. Briefly, this study shows that K_D-la_ directly driven by d-lactate exists as a regulatory mechanism and provides insights into the functional roles of d-lactate in prokaryotes.

Lysine l-lactylation (K_L-la_), a newly identified post-translational modification (PTM), has been found to be widely present in eukaryotes and prokaryotes (1, 2). Growing evidence suggests that K_L-la_ plays a key role in various physiological and pathological processes (3, 4, 5, 6, 7, 8, 9). Although lysine lactylation was initially reported to be generated by the l-enantiomer of lactate, lysine d-lactylation (K_D-la_), as the chiral counterpart, also exists in eukaryotic cells, with its precursor being a glycolytic metabolite (10). l-lactate, the end product of glycolysis, is converted from pyruvate by l-lactate dehydrogenase A (LdhA) and serves as a precursor driving the formation of K_L-la_ (11, 12). Moreover, hypoxia and the Warburg effect induce the expression and activity of LdhA, thereby resulting in extensive accumulation of l-lactate (13, 14). In contrast, its chiral enantiomer d-lactate exists at extremely low concentrations (11–70 nM), whereas l-lactate commonly reaches millimolar levels in human cells (15). d-lactate, a byproduct of glycolysis, is primarily generated from methylglyoxal *via* the glyoxalase pathway (16). S-d-lactoylglutathione, a high-energy intermediate of this pathway, can nonenzymatically react with lysine residues to form K_D-la_ (10, 17). The significant difference in cellular concentrations between l-lactate and d-lactate suggests that proteins are predominantly modified by l-lactylation in eukaryotes. Recently, Zhang *et al.* (18) developed a methodology to distinguish lysine lactylation isomers, demonstrating that K_L-la_ is the major form responsive to glycolysis and the Warburg effect. In addition, it is found that K_L-la_, rather than K_D-la_, is the dominant lysine lactylation induced by hypoxia (19).

In contrast to eukaryotes, prokaryotes may produce and accumulate both l-lactate and d-lactate because of the vast species diversity and metabolic heterogeneity (20, 21, 22). Although glycolysis serves as a fundamental and conserved metabolic pathway in organisms, its terminal step mediated by LdhA displays stereochemical divergence between eukaryotes and some prokaryotes, suggesting the distinct evolutionary adaptations in lactate metabolism (23, 24). In eukaryotes, the LdhA/B exclusively produces l-lactate (18). By contrast, the LdhA catalyzes the conversion of pyruvate into the enantiomeric d-lactate in many prokaryotes (25, 26, 27). The stereospecific activity of different LdhA toward pyruvate determines the chirality of the resulting lactate. Our previous work demonstrated the presence of l-lactylation in prokaryotes (2). However, it remains unclear whether d-lactate can serve as a precursor to drive the generation of K_D-la_ and whether K_D-la_ can reflect the dynamic changes in cellular glycolysis.

Here, we reported that d-lactate-derived d-lactylation on lysine residues serves as a PTM (K_D-la_) in prokaryotes. We revealed that acetate CoA-transferase (YdiF) can catalyze the production of d-lactyl-CoA, which serves as the d-lactyl donor, connecting d-lactate to K_D-la_ formation. Notably, we identified 86 K_D-la_ sites on 71 proteins in *Escherichia coli*. In addition, the data demonstrated that the *E. coli* enhances glycolysis in response to anaerobic conditions, which stimulates the production of d-lactate *via* LdhA and increases the K_D-la_ level. We also found that CobB functions as an endogenous de-d-lactylase. Furthermore, the results showed that K257 of glyceraldehyde-3-phosphate dehydrogenase (GapA) is important for bacterial growth. Our data suggest that K_D-la_ directly driven by d-lactate exists in prokaryotes as a regulatory mechanism linked to metabolic adaptation.

## Results

### Existence of the lysine d-lactylation in *E. coli*

**Figure 1:**
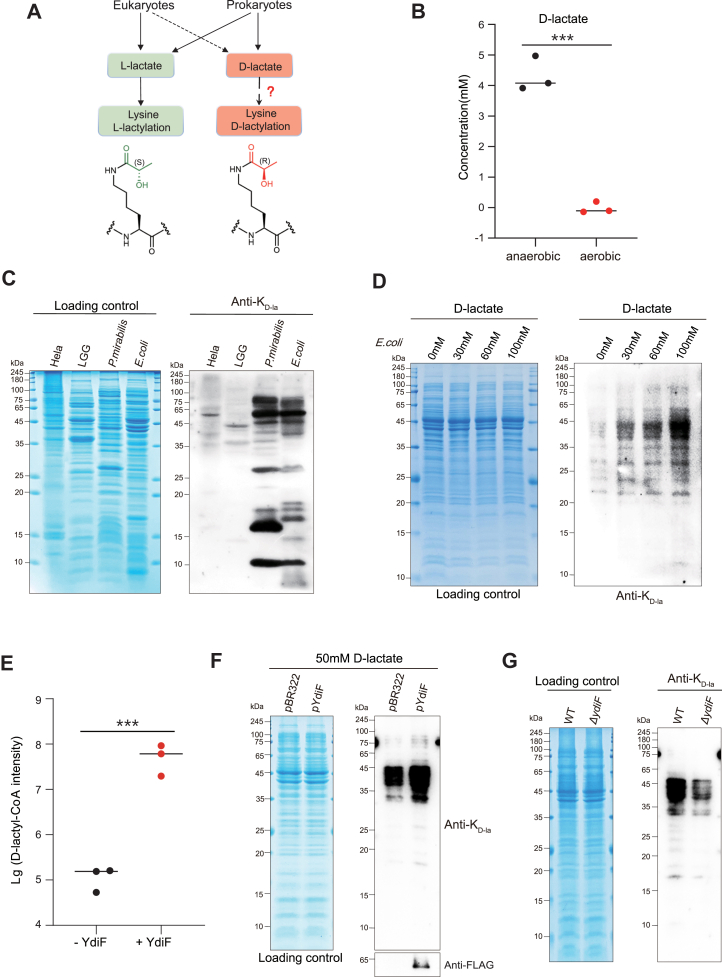
**Existence of the****d****-lactate-derived K_D-la_.***A*, differences in the chiral forms of lactate between prokaryotes and eukaryotes and potential mechanisms of the K_D-la_ formation. *B*, analysis of d-lactate levels. *Escherichia coli* WT cells were cultured overnight in LB medium under aerobic and anaerobic conditions at 37 °C. The supernatants were collected and analyzed using the d-Lactate Assay Kit. *n* = 3 biological repetitions. *C*, immunoblotting analyzed the K_D-la_ levels on whole-cell lysates using a pan anti-K_D-la_ antibody from human cervical cancer cells (HeLa), *Lactobacillus rhamnosus* (LGG), *Proteus mirabilis*, and *E.coli*. *D*, exogenous d-lactate enhances K_D-la_ levels. *E. coli* WT cells were cultured in LB medium supplemented with 0, 30, 60, and 100 mM sodium d-lactate until the bacterial absorbance reached 0.4 to 0.5 at 600 nm at 37 °C under aerobic conditions. K_D-la_ levels on whole-cell lysates were analyzed by immunoblotting. *E*, YdiF catalyzes the generation of d-lactyl-CoA. Reactions containing d-lactate and acetoacetyl-CoA (Acac-CoA) were performed in the presence or the absence of YdiF. d-lactyl-CoA was quantified by HPLC–MS/MS. n = 3 biological repetitions. *F*, immunoblotting analyzed K_D-la_ levels of *E. coli* MG1655 transferred pBR322 as control and *ydiF*-pBR322 as overexpressing strains (pYdiF). *G*, deficiency of YdiF decreased K_D-la_ in *E. coli*. K_D-la_ levels of Δ*ydiF* strain were analyzed by immunoblotting, with *E. coli* WT strain as control. All immunoblots had three biological repetitions, with similar results. Data are presented as means ± SD from three independent assays. Statistical significance was determined by a two-tailed Student’s *t* test (*p* < 0.05). K_D-la_, lysine d-lactylation.

Although both lactate enantiomers are present in organisms, and l-lactate exerts its biological regulatory functions through K_L-la_, the nonmetabolic functions of its enantiomer, d-lactate, remain unknown (Fig. 1*A*) (1). To validate the production of d-lactate in *E. coli* wildtype, we cultured the bacteria overnight under aerobic and anaerobic conditions, collected the supernatants, and detected d-lactate levels using the d-Lactate Assay Kit. The results showed that the supernatant under anaerobic conditions contains over 3 mM of d-lactate, whereas it was nearly undetectable under aerobic conditions (Fig. 1*B*). We speculated that d-lactate may be efficiently metabolized under aerobic conditions. The data indicate that *E. coli* produces d-lactate in response to anaerobic conditions, similar to eukaryotes. Based on the results, we hypothesized the existence of lysine d-lactylation derived from d-lactate in *E. coli*.

To investigate the presence of K_D-la_, we carried out Western blot assay on whole-cell protein lysates using a pan anti-D-lactyllysine antibody from human cervical cancer cells (HeLa), *Lactobacillus rhamnosus* (LGG), *Proteus mirabilis*, and *E. coli*. As shown in Figure 1*C*, K_D-la_ is present in a variety of organisms, with distinct modification levels and distribution patterns. We also observed that the global levels of K_D-la_ in *P. mirabilis* and *E. coli* were significantly higher than those in HeLa and LGG cells. It is well known that eukaryotic organisms generate only trace levels of d-lactate, mainly *via* methylglyoxal metabolism, and LGG, as a Gram-positive bacterium, primarily produces l-lactate (15, 28, 29, 30, 31, 32). However, both *P. mirabilis* and *E. coli* are Gram-negative bacilli, and they possess LdhA that produce d-lactate.

Eukaryotic cells treated with exogenous l-lactate exhibit a significant increase in K_L-la_ levels, suggesting that K_L-la_ originates from l-lactate (1). Therefore, to further investigate the relationship between d-lactate and K_D-la_, we cultured *E. coli* wildtype cells with supplementation of 0, 30, 60, and 100 mM sodium d-lactate until the bacterial absorbance at 600 nm reached 0.4 to 0.5. Indeed, Western blot analysis showed that K_D-la_ status in *E. coli* whole-cell lysate was significantly altered in a dose-dependent manner (Fig. 1*D*). In contrast, K_L-la_ levels showed no significant changes under d-lactate treatment conditions compared with the control (Fig. S1*B*). Our results showed that K_D-la_ was enhanced by treatment with sodium d-lactate, suggesting that d-lactate is a key determinant of K_D-la_.

### YdiF serves as a d-lactyl-CoA transferase

Recent studies have revealed that l-lactyl-CoA, derived from l-lactate, serves as a critical precursor for the formation of K_L-la_ (6). Our previous study demonstrated that YidF exhibits l-lactyl-CoA transferase activity (2). To investigate whether YdiF can catalyze the conversion of d-lactate to d-lactyl-CoA, we incubated purified YdiF with d-lactate *in vitro*, followed by analysis using LC–MS/MS. The result showed that YdiF efficiently catalyzed the production of d-lactyl-CoA from d-lactate compared with the control (Figs. 1*E* and S1, *A* and *C*). To further validate the enzymatic activity of YdiF, we constructed the *E. coli* MG1655 strain overexpressing YdiF (pYdiF) and the control strain harboring the empty pBR322 vector. The bacteria were cultured in LB medium with 50 mM sodium d-lactate. Western blot analysis using an anti-FLAG antibody confirmed the expression of YdiF in the pYdiF strain. Consequently, the pYdiF strain showed a marked increase in K_D-la_ levels compared with the control (Fig. 1*F*). Furthermore, we cultured *E. coli* wildtype strain and *ydiF* knockout *E. coli* strain (Δ*ydiF*). Immunoblotting analysis showed a significant reduction in K_D-la_ levels in the Δ*ydiF* strain compared with the wildtype strain (Fig. 1*G*). Together, our results demonstrate that YdiF functions as a d-lactyl-CoA transferase and reveal the mechanism of the K_D-la_: d-lactate is converted into the d-lactyl-CoA through YdiF, which can in turn be used by cells for lysine d-lactylation reactions.

### Identification and validation of lysine d-lactylated proteins in *E. coli*

**Figure 2:**
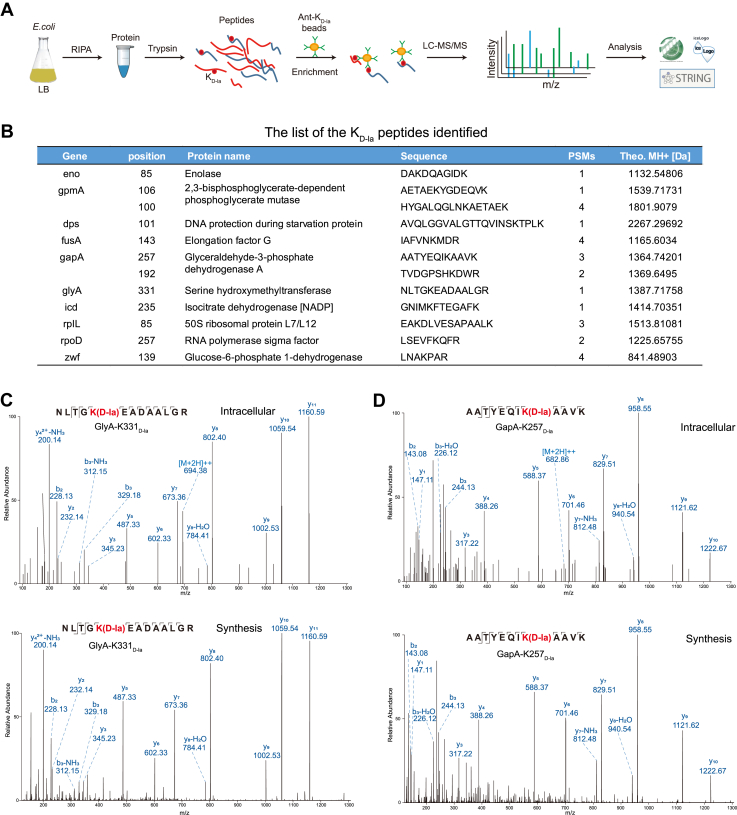
**Identification and validation of lysine****d****-lactylated peptides in *Escherichia coli*.***A*, flowchart showing the experimental procedure for the analysis of lysine d-lactylome. *B*, list of K_D-la_ peptides identified by LC–MS/MS from *E. coli* cell. *C* and *D*, the MS/MS spectra of the intracellularly modified peptides (NLTGK(D-la)EADAALGR, GlyA) and (AATYEQIK(D-la)AAVK, GapA), compared with their synthetic counterparts. K_D-la_, lysine d-lactylation.

To further identify potential lysine d-lactylation proteins, we affinity enriched lysine d-lactylation containing peptides as previously reported (2). Following the proteomics workflow (Fig. 2*A*), the K_D-la_ peptides were enriched with anti-K_D-la_ antibody from the tryptic digest peptides and then analyzed by nano-HPLC/MS/MS. A careful manual verification was performed to ensure accurate peptide identification. We first identified 86 K_D-la_ sites in 71 proteins in wildtype *E. coli* strain (Fig. 2*B* and Table S2). To determine whether the observed mass shift of 72.021 Da represented d-lactylation, we synthesized four K_D-la_ peptides of varying lengths, each bearing the same sequence as *in vivo*. The resulting peptides were analyzed in pairs by HPLC–MS/MS. For example, the entire MS/MS spectra of the intracellularly modified peptides from *E. coli* serine hydroxymethyltransferase K331 (NLTGK(D-la)EADAALGR) and GapA K257 (NLTGK(D-la)EADAALGR) matched almost completely with those of the synthetic counterparts. All the MS/MS with the detailed peak assignments are shown in Figures 2, *C* and *D* and S2, *A* and *B*. These evidences further confirmed the presence of K_D-la_ and the reliability of the identified K_D-la_ peptides.

### Functional annotation analysis of *E. coli*d-lactylome

**Figure 3:**
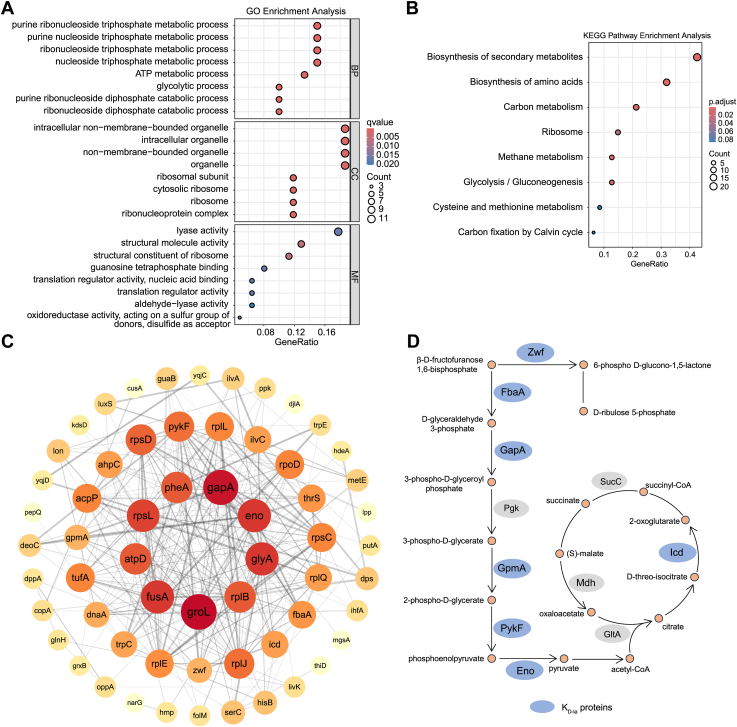
**Functional annotation and protein–protein interaction analysis of lysine****d****-lactylated proteins in *Escherichia coli*.***A*, representative Gene Ontology (GO) annotations of K_D-la_ proteins for Biological Process (BP), Cellular Component (CC), and Molecular Function (MF). *B*, KEGG pathway analysis for K_D-la_ proteins. *C*, visualization and enrichment of the 53 highly interconnected protein networks (listed by gene names) were performed using the STRING database and Cytoscape software. *D*, glycolysis and tricarboxylic acid pathway proteins with lysine d-lactylation in *E. coli*. Proteins without lysine d-lactylation were indicated in *gray*. Benjamini and Hochberg correction was used to adjust *p* values. K_D-la_, lysine d-lactylation; KEGG, Kyoto Encyclopedia of Genes and Genomes.

To further investigate the potential function of K_D-la_, we conducted functional enrichment analyses of the identified K_D-la_ substrates. First, protein class analysis revealed that over 50% of K_D-la_ substrates were categorized as metabolite interconversion enzymes, 18.2% as translational proteins, and 12.7% as transporters (Fig. S3*A*). Furthermore, we carried out functional annotation analysis against the Gene Ontology (GO) and Kyoto Encyclopedia of Genes and Genomes (KEGG) pathway. The GO enrichment analysis showed that the d-lactylated proteins were significantly associated with nucleic acid metabolism, ATP metabolism, glycolysis, and ribosome functions (Fig. 3*A*). In the KEGG metabolic pathway analysis, biosynthesis of secondary metabolites, biosynthesis of amino acids, carbon metabolism, and ribosomes were among the most highly enriched pathways (Fig. 3*B*).

To evaluate the conserved substrate motifs of d-lactylated residues, we analyzed the amino acids adjacent to the d-lactylated lysine from −7 to +7 using iceLogo software (Ghent University) . The results revealed that alanine and proline were over-represented at +1 position. Lysine and arginine were also over-represented at the +3 position near the K_D-la_ sites. In addition, lysine and aspartic acid showed relatively high abundance at the −5 position (Fig. S3*B*).

Protein–protein interactions are pivotal to the function and regulation of cellular physiology. To investigate the correlation of d-lactylated proteins in *E. coli*, we analyzed the protein–protein interaction networks *via* STRING and Cytoscape. The results revealed that a cluster of 53 d-lactylated proteins was highly interconnected, and these proteins were functionally associated with ribosome, glycolysis, and translation factor activity (Fig. 3*C*). These enrichment analyses of K_D-la_ proteins revealed that they are predominantly distributed in metabolism and biosynthesis, particularly glycolysis, which suggests that K_D-la_ may play a role in regulating energy metabolism (Fig. 3*D*).

### Dynamics of the K_D-la_ in response to anaerobic stress and energy conditions by LdhA

**Figure 4:**
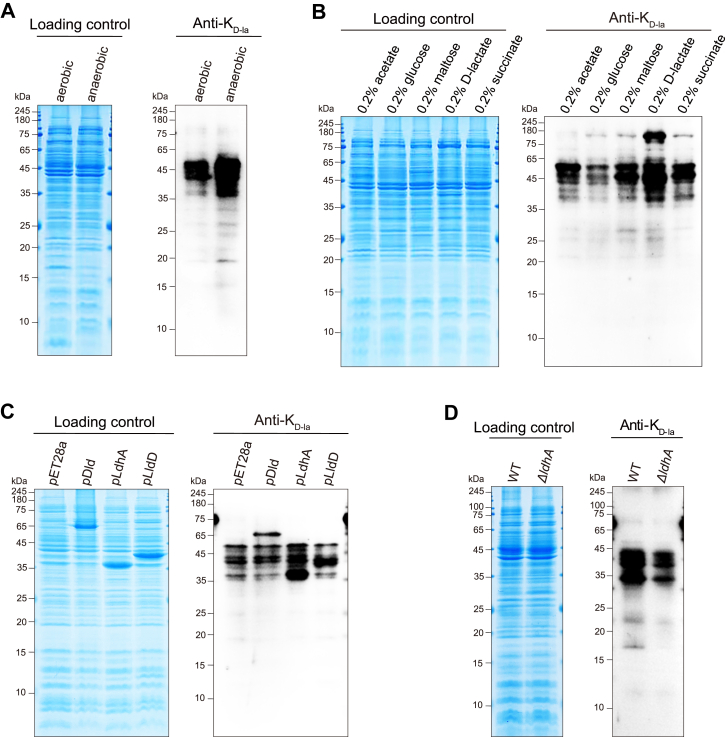
**Dynamics of K_D-la_ in response to energy conditions.***A*, immunoblotting analysis of K_D-la_ levels on whole-cell protein lysates from *Escherichia coli* WT strain cultured overnight in LB medium under anaerobic or aerobic conditions at 37 °C. *B*, *E. coli* WT cells were cultured overnight in M9 medium supplemented with five carbon sources: 0.2% acetate, 0.2% glucose, 0.2% d-lactate, 0.2% maltose, or 0.2% succinate. K_D-la_ levels on whole-cell lysates were analyzed by immunoblotting using a pan anti-K_D-la_ antibody. *C*, *E. coli* BL21 (λDE3) was transferred with the empty vector (pET28a) as a control and with vectors *ldhA*-pET28a, *lldD*-pET28a, and *dld*-pET28a as overexpressing strains (pDld, pLldD, and pLdhA), the K_D-la_ levels were analyzed by immunoblotting. *D*, deficiency of LdhA decreased K_D-la_ in *E. coli*. K_D-la_ levels of Δ*ldhA* strain were analyzed by immunoblotting, with *E. coli* WT strain as a control. All immunoblots had three biological repetitions, with similar results. K_D-la_, lysine d-lactylation.

The extracellular d-lactate can enhance K_D-la_, leading us to hypothesize that modulation of intracellular d-lactate production would affect K_D-la_ levels. Our data have shown that *E. coli* exhibits increased glycolysis and d-lactate production under anaerobic conditions. Therefore, we performed a Western blotting analysis to assess K_D-la_ levels in protein lysates of *E. coli* grown in anaerobic or aerobic LB medium. The results demonstrated that anaerobic conditions induced a significant and extensive increase in K_D-la_ levels (Fig. 4*A*). The finding revealed that K_D-la_ is a type of PTM derived from d-lactate in response to anaerobic stress.

K_D-la_ can be stimulated by d-lactate, which is an energy source, and can be regulated by carbon sources in bacteria (33, 34). Therefore, we further examined the effect of different carbon sources on the K_D-la_ status. We cultured *E. coli* wildtype cells in M9 medium and treated them with five key metabolic intermediates with 0.2% acetate, 0.2% glucose, 0.2% maltose, 0.2% d-lactate, and 0.2% succinate, respectively. Immunoblotting analysis showed that various carbon sources markedly modulate K_D-la_ levels in *E. coli* (Fig. 4*B*). Although the mechanisms by which different carbon sources regulate K_D-la_ remain unclear, our data establish a link between the metabolic processes and lysine d-lactylation. Together, our findings revealed the dynamics of K_D-la_ levels under stress and varying nutrient conditions in *E. coli*.

Interestingly, it is known that *E. coli* possesses three LdhAs: Quinone-dependent d-lactate dehydrogenase (Dld) and l-lactate dehydrogenase (LldD) are respiratory enzymes that catalyze the oxidation of d-lactate and l-lactate to pyruvate, whereas LdhA converts pyruvate to d-lactate in the cytoplasm. We reasonably hypothesized that LdhA regulates d-lactate metabolism in a manner analogous to the regulation of l-lactate, thereby affecting the dynamics of K_D-la_ levels in *E. coli*. Therefore, we performed Western blotting to analyze K_D-la_ levels in *E. coli* strains overexpressing *dld*, *lldD*, and *ldhA* (pDld, pLldD, and pLdhA). Compared with the *E. coli* strain harboring pET28a, we observed that only the pLdhA strain exhibited a significant increase in K_D-la_ levels (Fig. 4*C*). Furthermore, we cultured *E. coli* wildtype strain and *ldhA* knockout *E. coli* strain (Δ*ldhA*). Western blotting assay showed that the K_D-la_ levels in the Δ*ldhA* strain were significantly decreased compared with the wildtype strain (Fig. 4*D*). In addition, we measured the d-lactate levels in both strains following overnight anaerobic culture. The results showed that d-lactate in Δ*ldhA* strain was almost undetectable in the supernatant (Fig. S4). These findings demonstrate that LdhA regulates K_D-la_ levels by modulating d-lactate metabolism.

### CobB mediates de-d-lactylation in *E. coli*

**Figure 5:**
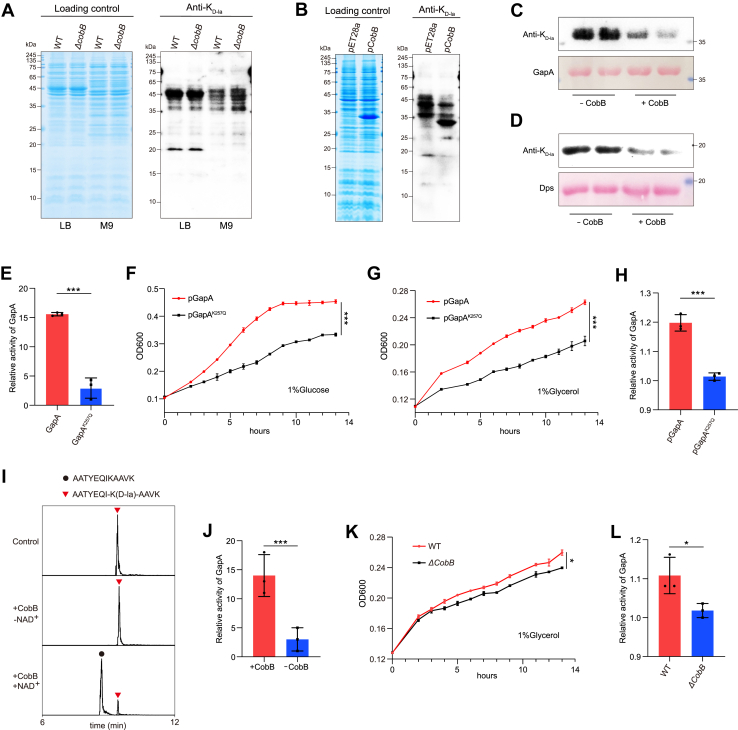
**The K257_D-la_ of GapA affects *Escherichia coli* growth.***A*, *E. coli* WT strain and Δ*cobB* strain were cultured overnight at 37 °C in LB or M9 medium, and K_D-la_ levels on whole-cell protein lysates were analyzed by immunoblotting. *B*, immunoblotting analysis of K_D-la_ levels of *E. coli* BL21 (λDE3) transferred pET28a as control and *cobB*-pET28a as overexpressing strains (pCobB). *C* and *D*, recombinant GapA and Dps were incubated with CobB at 37 °C for 2 h, and K_D-la_ levels were assessed by immunoblotting. *E*, detection of the activities of recombinant proteins, GapA and GapA^K257Q^. *F* and *G*, growth curves of pGapA and pGapA^K257Q^ strains cultured in M9 medium (with 1% glucose or 1% glycerol) at 37 °C were monitored in 96-well plates by measuring absorbance at 600 nm. *H*, measurement of the intracellular GapA activity of pGapA and pGapA^K257Q^ strains related to *F* and *G*. *I*, CobB catalyzes de-d-lactylation of GapA K257_D-la_ peptide *in vitro*. MS1 extracted ion chromatograms (XICs) of the d-lactylated peptide (NLTGK(D-la)EADAALGR) and the corresponding de-d-lactylated product after incubation with CobB are shown. The GapA K257_D-la_ peptide was incubated with CobB, and reaction products were analyzed by LC–MS/MS. *J*, CobB regulation GapA activity by erasing K257_D-la_*in vitro*. Recombinant GapA was incubated with CobB to decrease K257_D-la_, after de-d-lactylation, detection of the activity of GapA. *K*, measurement growth curve of *E. coli* WT strain and Δ*cobB* strain cultured in M9 medium with 1% glycerol at 37 °C. *L*, measurement of the intracellular activity of GapA of strains related to *K*. All immunoblots had three biological repetitions, with similar results. Data are presented as means ± SD from three independent assays. Statistical significance was determined by a two-tailed Student’s *t* test (*p* < 0.05). K_D-la_, lysine d-lactylation.

It is well established that NAD-dependent protein deacylase (CobB) is the main deacylase for the removal of acetylation, succinylation, and 2-hydroxyisobutyrylation in *E. coli* (35, 36, 37). Our recent research also found that CobB functions as an endogenous de-l-lactylase (2). To examine the de-d-lactylation activity of CobB in *vivo*, we cultured *E. coli* wildtype strain and *cobB* knockout *E. coli* strain (Δ*cobB*) in LB and M9 media, respectively. Western blotting analysis showed that Δ*cobB* strain showed no change in K_D-la_ levels compared with wildtype strain in LB medium, whereas the K_D-la_ levels were slightly increased in Δ*cobB* strain when cultured in M9 medium (Fig. 5*A*). Furthermore, we constructed a CobB-overexpressing *E. coli* BL21 strain (pCobB). Compared with *E. coli* harboring the pET28a vector, the pCobB strain caused a significant decrease in K_D-la_ levels (Fig. 5*B*). These results proved that CobB can remove d-lactylation in *vivo*.

To further validate the enzymatic activity of CobB toward K_D-la_ *in vitro*, we selected two candidate substrates based on the d-lactylome: DNA protection during starvation protein (Dps) and GapA. We expressed and purified the CobB, Dps, and GapA proteins and further incubated recombinant Dps or GapA with CobB in the presence of NAD^+^. We then detected K_D-la_ levels of candidate proteins by immunoblotting. The results showed that the K_D-la_ levels of Dps and GapA decreased significantly in the presence of CobB (Fig. 5, *C* and *D*). These data indicated that CobB catalyzes the removal of lysine-d-lactylation *in vitro*. It was further confirmed that CobB functions as a de-d-lactylase.

### Effect of GAPA K257_D-la_ on bacteria growth

Functional annotation analysis demonstrated significant enrichment of K_D-la_ proteins in cellular metabolic processes, particularly in the glycolytic pathway (Fig. 3*D*). To further investigate the function of K_D-la_ in metabolic regulation, we focused on GapA, a conserved key glycolytic enzyme. We have confirmed the reliability of the identified K_D-la_ peptides containing GapA K257_D-la_ *via* synthetic peptides (Fig. 2*D*). Furthermore, multiple sequence alignment of GapA homologs across five different species from bacteria to humans showed that the GapA lysine 257 was completely conserved (Fig. S5*A*). To determine the importance of K257 within GapA and its effects on enzymatic activity, we mutated lysine 257 to glutamine (GapA^K257Q^), which structurally mimics lysine d-lactylation by neutralizing the positive charge under physiological conditions. In combination with *in vitro* enzymatic activity assays, we found that the enzymatic activity of the GapA^K257Q^ variant was significantly reduced compared with that of the wildtype protein (Fig. 5*E*). This result suggests that K257_D-la_ influences the activity of GapA. To further investigate whether GapA K257_D-la_ influences bacterial growth, we constructed *E. coli* MG1655 strains (pGapA and pGapA^K257Q^) overexpressing GapA or GapA^K257Q^. GapA catalyzes the oxidative phosphorylation of G3P to 1,3-bisphosphoglycerate. Considering that G3P can be produced from both glucose and glycerol metabolism, we cultured the strains in M9 minimal medium supplemented with either 1% glucose or 1% glycerol as the sole carbon source. Growth curves of the pGapA and pGapA^K257Q^ strains were subsequently measured. We observed that the pGapA^K257Q^ strain exhibited significantly slower growth than the pGapA strain under both carbon source conditions with comparable levels of protein overexpression (Figs. 5, *F* and *G* and S5*C*). Furthermore, intracellular GapA enzymatic activity was assessed. Consistent with the growth phenotypes, the pGapA^K257Q^ strain displayed reduced activity compared with the pGapA strain (Fig. 5*H*). These results indicate that GapA K257 influences bacterial growth.

*In vitro* assays showed that CobB can remove d-lactylation on GapA. To further confirm this observation, we incubated the synthetic GapA K257_D-la_ peptide (NLTGK(D-la)EADAALGR) with CobB, and the reaction products were analyzed by LC–MS/MS. The results showed that CobB catalyzed the de-d-lactylation of the GapA K257 peptide *in vitro* (Figs. 5*I* and S5*D*). Then, GapA protein was incubated with CobB to erase the K257 d-lactylation, followed by enzymatic activity measurements. The results showed that CobB-mediated removal of the K_D-la_ of GapA significantly enhanced GapA enzymatic activity *in vitro* (Fig. 5, *C* and *J*). In addition, we measured the growth curves and intracellular GapA activity of the *E. coli* wildtype strain and Δ*cobB* strain cultured in M9 minimal medium supplemented with 1% glycerol. The data showed that wildtype strain exhibited faster growth than the Δ*cobB* strain (Fig. 5*K*). Consistently, intracellular GapA activity in the wildtype strain was higher than that in the Δ*cobB* strain (Fig. 5*L*). Together, these results indicate that CobB regulates GapA enzymatic activity and bacterial growth by erasing GapA K257_D-la_ both *in vitro* and *in vivo*.

## Discussion

As a chiral metabolite, lactate exists in two enantiomers: l-lactate and d-lactate. In eukaryotes, l-lactate predominates as the major form, with its concentration exceeding d-lactate by four orders of magnitude (15, 38). The crucial roles of l-lactate in energy metabolism and cellular signaling have been progressively elucidated, whereas the emerging evidence highlights the biological significance of d-lactate (12, 39, 40, 41, 42). Recent studies suggest that d-lactate not only functions as an immunogen to enhance antitumor immunity by polarizing hepatic macrophages from the M2 to the M1 phenotype, but also synergizes with l-lactate to activate mitochondrial electron transport chains, stimulate pyruvate oxidation, and serve as a metabolic signaling molecule (39, 41, 43). These findings reveal that d-lactate as a previously overlooked metabolite may play a role in modulating physiological processes. Notably, mammalian d-lactate is minimally produced endogenously and primarily originates from gut microbiota metabolism. Clinical observations reveal significantly elevated plasma d-lactate levels in rare conditions, including certain cases of short bowel syndrome, strongly correlating with gut dysbiosis (16, 44). These phenomena suggest that d-lactate may serve as a critical cross-talk molecule mediating host–microbiota metabolic interactions. Although d-lactate participates in bacterial energy metabolism, its nonmetabolic functions remain poorly understood. Therefore, systematic investigation into the regulatory and functional mechanisms of d-lactate in microbial physiology will advance our understanding of how gut microbial metabolites modulate host health.

**Figure 6:**
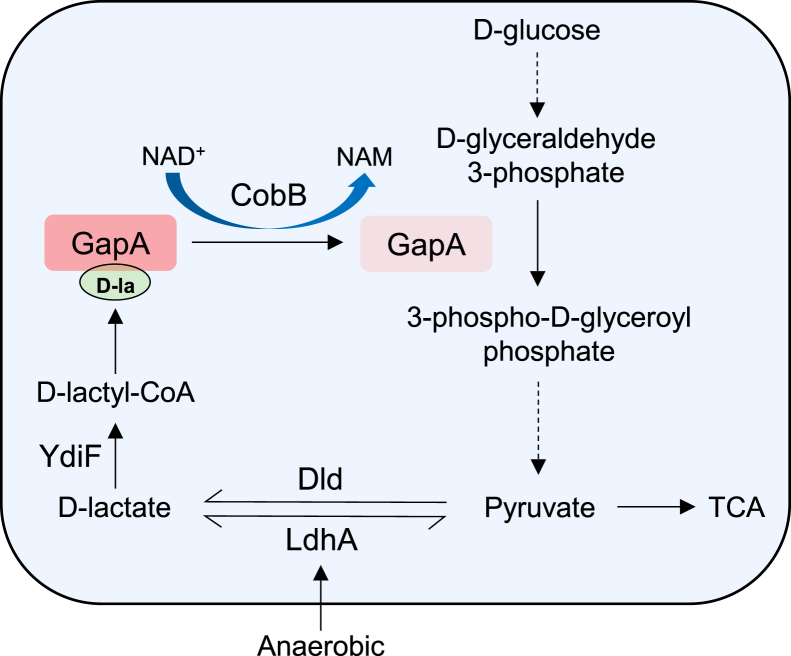
**Graphic model as discussed in the text.** Glycolysis-derived d-lactate drives K_D-la_ formation that regulates bacterial growth. K_D-la_, lysine d-lactylation.

In this study, we demonstrate that *E. coli* produces d-lactate *via* the glycolytic pathway, supporting the biological plausibility of d-lactate-driven lysine d-lactylation in prokaryotes (Fig. 6). We identified K_D-la_ that can be stimulated by exogenous d-lactate in *E. coli*. These findings reveal the chiral divergence in metabolic regulation between prokaryotes and eukaryotes, offering novel evolutionary perspectives for understanding the functional roles of chiral molecules in organisms. While previous studies found the presence of K_D-la_ in eukaryotes through nonenzymatic reactions between S-d-lactoylglutathione and lysine residues, our discovery of a d-lactate-dependent K_D-la_ pathway is a canonical acylation mechanism, representing a fundamentally distinct mechanism. Furthermore, we revealed that YdiF catalyzes d-lactyl-CoA formation, thereby bridging d-lactate metabolism with K_D-la_ modification. Notably, our prior work demonstrated that YdiF exhibits broad substrate diversity *in vitro*, generating l-lactyl-CoA and acetyl-CoA with nearly equivalent catalytic efficiency for acetate, l-lactate, and d-lactate, suggesting its potential role as a central regulatory hub governing bacterial acyl-CoA homeostasis.

Notably, we found LdhA as the key regulator of d-lactate production, with in *vivo* d-lactate fluctuations directly impacting K_D-la_ levels. Anaerobic-induced glycolysis increases, and subsequent d-lactate accumulation significantly elevates global K_D-la_ modification levels, mirroring the metabolic regulation of l-lactate and its effects on K_L-la_ modification in eukaryotes. This functional parallelism suggests that K_D-la_ may play critical roles in prokaryotic cellular activities.

We identified 86 K_D-la_ sites on 71 proteins in *E. coli*, validated by synthetic peptides combined with HPLC–MS/MS. These modified proteins predominantly localize to glycolysis and the tricarboxylic acid cycle, suggesting that d-lactate derived *via* LdhA may feedback regulate enzymatic activity within the glycolytic pathway through K_D-la_. It should be noted that the stoichiometry of the K_D-la_ at individual sites remains unclear, and the apparent enrichment of glycolytic and tricarboxylic acid cycle enzymes may partially reflect their high cellular abundance. Importantly, our functional analyses demonstrate that d-lactylation at K257 of GapA suppresses bacterial growth. Moreover, we identified CobB as the de-d-lactylase *in vitro* and in *vivo*. Importantly, CobB regulates GapA K_D-la_ levels and is associated with the observed growth phenotypes, suggesting the potential functional mechanism of K_D-la_ in bacterial metabolic regulation. The identification of lysine d-lactylation and its regulatory enzyme CobB represents a crucial foundation for future exploration of other regulatory enzymes and characterization of biological functions of K_D-la_.

## Experimental procedures

### Strains and reagents

*E. coli* MG1655 cells and *E. coli* Δ*cobB* cells were obtained from our previous study (2), *E. coli* BL21 (λDE3) was from TIANGEN. The *E. coli* Keio Parent Strain *BW25113*, Δ*ydiF* and *ΔldhA* cells were sourced from an *E. coli* single-gene deletion mutant library (Keio collection). The *ydiF* and *cobB* genes overexpressing *E. coli* BL21 (λDE3) strains were obtained from our previous study (2). All strains were grown in LB broth at 37 °C in shaken flasks at 220 rpm overnight under aerobic conditions, unless otherwise noted. Sequencing-grade trypsin was purchased from Beijing Shengxia Proteins Scientific Ltd. The anti-d-lactyllysine antibodies were purchased from PTM Biolabs Inc. All the synthetic peptides were generated by Synpeptide Co Ltd. Other reagents are listed in Table S6.

### Construction of genes overexpressing *E. coli* strains

This method was performed as described previously (2). We used pEASY-Basic Seamless Cloning and Assembly Kit (TRANS) to construct the plasmids of pET28a-ldhA, pET28a-dld, pET28a-lldD, pET28a-dps, pET28a-gapA, and pBR322-gapA-FLAG tag. For mutation, we used the Fast Mutagenesis System (TRANS) to construct the vectors containing point-mutated genes. The primers used for PCR are listed in Table S6. The constructed vectors of pET28a were transformed into *E. coli* BL21 (λDE3) for overexpressing proteins. The constructed vectors of pBR322 were transformed into *E. coli* MG1655 and cultured in LB medium containing ampicillin (50 μg ml^–1^), at 37 °C in shaken flasks at 220 rpm overnight.

### Western blot

Cells were harvested and lysed in lysis buffer supplemented with protease inhibitor cocktail, and protein concentrations were determined using a bicinchoninic acid assay. Equal amounts of protein were separated by SDS-PAGE and transferred onto polyvinylidene fluoride membranes. Membranes were blocked with 5% (w/v) nonfat milk in Tris-buffered saline with Tween-20 for 2 h at room temperature and then incubated with primary antibodies at 4 °C overnight. The membranes were incubated with horseradish peroxidase–conjugated secondary antibodies for 1 h at room temperature. Signals were detected using an enhanced chemiluminescence substrate and visualized with a chemiluminescence imaging system.

### d-lactate concentrations detection

*E. coli* wildtype cells and Δ*ldhA* cells were cultured overnight in LB medium under aerobic and anaerobic conditions at 37 °C. The supernatants were collected by high-speed centrifugation for 15 min, and then, the concentrations of the secreted d-lactate using a d-Lactate Assay Kit (Elabscience) were analyzed. Each data were performed with three biological repetitions.

### Purification of recombinant proteins

Transformed the gene-pET28a vectors into the *E. coli* BL21 (λDE3) and cultured them in LB medium with kanamycin (50 μg ml^−1^) at 37 °C and then shaken flasks until the absorbance was at 0.6 to 0.8 at 600 nm. Next, cells were stimulated by 0.05 mM IPTG at 16 °C overnight, followed by harvesting of whole-cell lysates for immunoblotting or protein purification. Cells were crushed by ultrasonication in lysis buffer (20 mM Tris–HCl, pH = 8.0, 10 mM MgCl_2_, 1 mg ml^−1^ lysozyme, and 50 U ml^−1^ nuclease). The whole-cell lysates were mixed with HisPur Nickel–Nitrilotriacetic Acid Resin and washed by wash buffer (20 mM Na_3_PO_4_, 300 mM NaCl, 25 mM imidazole, pH = 7.4), and the target proteins were eluted with elution buffer (20 mM Na_3_PO_4_, 300 mM NaCl, 250 mM imidazole, pH = 7.4). The elution buffer was collected and concentrated using an Amicon Ultra-0.5 Centrifugal Filter Device in PBS buffer.

### *In vitro* assay for YdiF activity

Assay of the YdiF CoA-transferase activity was performed as described previously (2). The enzyme reactions were carried out in phosphate buffer (50 mM NaH_2_PO_4_, 50 mM Na_2_HPO_4_, and 5 mM MgCl_2_, pH 7.0) containing 1 mM acetoacetyl-CoA and 10 mM d-lactate at 35 °C for 20 min and initialized by adding the 7.5 μg of purified YdiF. The total volume of the reaction mixture was 50 μl. The mixtures were preincubated at the designated reaction temperature for 5 min before the initiation. The reactions were terminated by adding an equal volume of 10% (v/v) trifluoroacetic acid and centrifugation, the supernatant was cleaned with C18 ZipTips, and the products of d-lactyl-CoA were analyzed by HPLC–MS/MS. Each reaction was performed with three biological repetitions.

### HPLC–MS/MS analysis for d-lactyl-CoA

Samples dissolved in water were analyzed by the Orbitrap Exploris 480 (Thermo Scientific) mass spectrometer in positive electrospray ionization mode using the settings described previously. In detail, the samples were separated by an ACQUITY BEH C18 column (2.1∗100 mm, 1.7 μm), and the HPLC gradient for 15 min was set up as follows: 0.2 ml/min flow at 98% buffer A (water with 5 mM ammonium acetate) and 2% buffer B (95% acetonitrile in water with 5 mM ammonium acetate) for 0 min, 98% to 70% buffer A for 8 min, 70% to 2% buffer A for 1 min, 2% buffer A for 3 min, 2% to 98% buffer A for 1 min, and 98% buffer A for 2 min. An Orbitrap Exploris 480 (Thermo Fisher Scientific) was employed for MS analysis. Spray voltage was set to 3.5 kV, funnel RF level at 40, and ion transfer tube temperature at 320 °C. Data were collected by Xcalibur (version 4.0.27.19). The normalized automatic gain control target and maximum injection time were set at 70%/standard for MS1. The orbitrap mass analyzer was used as the MS2 detector with 120,000. Targeted mass setting is d-lactyl-CoA: 840.1436. MS2 isolation window was 2 Da, and mass tolerance was 10 ppm, and a normalized higher-energy collision–induced dissociation collision energy of 25% was used for precursor fragmentation. The relative intensity of d-lactyl-CoA was analyzed by Xcalibur (version 4.0.27.19).

### Sample preparation and immunoaffinity enrichment

As described previously (2), *E. coli* MG1655 was cultured at 37 °C in LB medium overnight. Cells were harvested and sonicated on ice in radioimmunoprecipitation assay lysis buffer. After centrifugation (16,000*g*) at 4 °C for 20 min, the supernatant was collected. The 2 mg proteins were precipitated by trichloroacetic acid, precipitates were dissolved in 100 mM NH_4_HCO_3_ with overnight digestion by trypsin (trypsin:protein ratio, 1:50). Digested products were incubated with 5 mM DTT at 56 °C for 1 h, followed by alkylation with 15 mM iodoacetamide for 45 min at room temperature under darkness. Excess iodoacetamide was blocked with 30 mM cysteine, and the last digestion was performed at a ratio of 1:100 for 4 h. Products were desalted by SepPak C18 cartridges (Waters) and dried. Tryptic peptides were redissolved in NETN buffer (50 mM Tris–HCl [pH 8.0], 100 mM NaCl, 1 mM EDTA, and 0.5% Nonidet P-40) and incubated with anti-d-lactyl lysine antibody–conjugated agarose beads (PTM Biolabs) at 4 °C overnight, with gentle rotation. The incubated beads were washed three times with NETN buffer, twice with NETN buffer (50 mM Tris–Cl [pH 8.0], 100 mM NaCl, and 1 mM EDTA) and three times with water. Bound peptides were eluted three times with 1% trifluoroacetic acid. Finally, the eluates were dried and cleaned with C18 ZipTips (Millipore Corp) before nano-HPLC–MS/MS analysis.

### HPLC–MS/MS analysis for K_D-la_

Enriched peptides were analyzed by HPLC–MS/MS. Samples were reconstituted in 0.1% formic acid and then injected into a nano-LC system (EASY-nLC 1200; Thermo Fisher Scientific). Peptides were resolved by a 75 μm-i.d., 25 cm-long C18 column (3 μm, Dr Maisch GmbH) at a flow rate of 300 nl/min. Gradient elution was performed with 2% to 45% HPLC buffer B (0.1% formic acid in 80% acetonitrile) for 90 min then transition to 100% buffer B for 15 min and keep with 100% buffer B for 5 min. An Orbitrap Eclipse Tribrid mass spectrometer (Thermo Fisher Scientific) was employed for MS analysis. Spray voltage was set to 2.1 kV, funnel RF level at 40, and ion transfer tube temperature at 320 °C. Mass spectrometric analysis was carried out in data-dependent acquisition mode of the most intense precursors, and data were collected by Xcalibur (version 4.0.27.19). The Orbitrap mass analyzer was used as the MS1 detector with 60,000 resolution and scan range 350 to 1750 *m/z*. The normalized automatic gain control target and maximum injection time were set at 100%/20 ms for MS1 and 200%/30 ms for MS2. Precursor ions with charges of +2 to +5 were isolated for MS2, and dynamic exclusion time was set at 50 s. The MS2 isolation window was 1.6 Da, and a normalized higher-energy collision-induced dissociation energy of 30% was used for precursor fragmentation.

### Database search for K_D-la_

The raw data were searched against the *E. coli* K12 protein database (Proteome ID: UP000000625) using the SEQUEST HT search algorithm implemented in Proteome Discoverer (version 3.0). A trypsin-specific digestion was specified, allowing up to two missed cleavages. Carbamidomethylation of cysteine was set as a fixed modification, whereas d-lactylation on lysine, oxidation of methionine, and protein N-terminal acetylation were set as variable modifications. The precursor ion mass tolerance was set to 10 ppm, and the fragment ion mass tolerance was set to 0.02 Da. Peptide-spectrum matches were evaluated using Percolator, and identifications were filtered using a Percolator *q* value <0.005 and a Percolator posterior error probability <0.05. In addition, MS/MS spectra supporting modification sites were manually inspected to ensure spectral quality, and sites supported exclusively by low-confidence spectra were excluded, and duplicate sites were removed (All identified peptides and proteins are listed in Tables S7 and S8).

### Functional annotation analysis

The KEGG pathway and GO function in three categories BP (Biological Processes), CC (Cellular Component), and MF (Molecular Functions) pathway enrichment analyzed through the R/Bioconductor package “clusterProfiler” (version: 4.0). The *p* value cutoff of 0.1 was selected as the cutoff criteria.

### Motif analysis of K_D-la_ peptides

The d-lactylated peptides were used to analyze the consensus flanking sequence of lysine d-lactylation sites with iceLogo software (version 1.3, Ghent University)**.** Seven neighboring amino acid residues on each side of the K_D-la_ sites were selected as the positive set for analysis. The embedded Swiss-Prot “*E. coli* (strain K12)” was used as the negative set.

### Protein–protein interaction network analysis

The STRING database was used for enrichment analysis of lysine d-lactylated protein–protein interaction networks in *E. coli*. All interactions with medium confidence (0.4) were visualized in Cytoscape (version 3.10.3).

### De-d-lactylation reaction by CobB *in vitro*

The recombinant protein Dps, GapA, was purified from *E. coli*. As the endogenous proteins, the preparation is expected to contain a background level of the K_D-la_ acquired during host expression. For the enzymatic activity assay, the purified recombinant CobB (10 μg) and candidate substrate proteins (Dps, GapA, 15 μg) were incubated in CobB reaction buffer at 37 °C for 2 h. The reaction products were analyzed by Western blotting with the pan anti-d-lactylation antibody.

### Nano-HPLC assay for determination of the reacted peptides of CobB

This method was described previously (2). In detail, GapA K257_D-la_ peptides (50 μM) with 1 μM CobB were incubated in CobB reaction buffer (50 mM Tris–HCl, 137 mM NaCl, 2.7 mM KCl, 1 mM MgCl_2_, 1 mM DTT, and 1 mM NAD^+^ [pH 8.5]) for 2 h at 37 °C. After quenching the reaction with an equal volume of 10% (v/v) trifluoroacetic acid, the samples were spun for 10 min at 18,000*g* to separate the enzyme. Samples were separated by a 28-min HPLC gradient (linear gradient from 5% to 50% HPLC buffer B [0.1% formic acid in acetonitrile] for 3 min and then to 100% buffer B for 20 min and keeping 100% buffer B for 5 min). The raw data were analyzed by extracting MS1 ion chromatograms (extracted ion chromatograms) of the d-lactylated peptide (NLTGK(D-la)EADAALGR) and its delactylated form using Xcalibur software.

### Assay for GapA activity

*E. coli* cells were harvested from 5 ml cultures, and the enzymatic activities of purified GapA and GapA^K257Q^ proteins were determined. GapA activity was measured using a G3P Dehydrogenase Activity Assay Kit (Solarbio, BC2210) following the protocol from manufacturer.

### Drawing a growth curve

As previously described (2), the strains were cultured in LB medium overnight, then diluted 1:1000 in M9 medium supplemented with either 1% glucose or 1% glycerol as the sole carbon source, and incubated at 37 °C with 96-well plates, and each strain was added to three wells. For growth assays in LB medium, strains were inoculated into 15 ml tubes and cultured at 37 °C with shaking at 220 rpm. The absorbance at 600 nm at initiation was set as a blank and measured every 1 h using a spectrophotometer. All the growth dynamic curves were drawn using GraphPad Prism 8.0.2 (GraphPad Software).

## Data availability

All data needed to evaluate the conclusions in the article are present in the article and/or the supporting information. Additional data related to this article may be requested from the authors. The MS proteomics data have been deposited to the ProteomeXchange Consortium (http://proteomecentral.proteomexchange.org) *via* the iProX partner repository with the dataset identifier PXD062906.

## Supporting information

This article contains supporting information.

## Conflict of interest

The authors declare that they have no conflicts of interest with the contents of this article.
