# Supplementary material for: d-lactate drives lysine d-lactylation to regulate metabolism in Escherichia coli
Source: J Biol Chem. 2026 Mar 16;302(5):111374. doi: 10.1016/j.jbc.2026.111374 (PMC13090971; doi:10.1016/j.jbc.2026.111374)
Supplement: Supplementary Information [file mmc1.pdf]

Supplementary Information

Supplementary Tables

Supplementary Table 1. The data of D-lactate concentrations measured.

Supplementary Table 2. The D-lactylated peptides identified of *E. coli* MG1655.

Supplementary Table 3. The data of YdiF with D-lactate reaction in vitro.

Supplementary Table 4. The source data of growth curve.

Supplementary Table 5. The source data for the relative activity of GapA.

Supplementary Table 6. The list of reagents, bacterial strains, and plasmids used in this paper.

Supplementary Table 7. Full list of peptides identified.

Supplementary Table 8. Full list of proteins identified.

Supplementary Table 2, 4, 5, 7 and 8 is available in the data file.

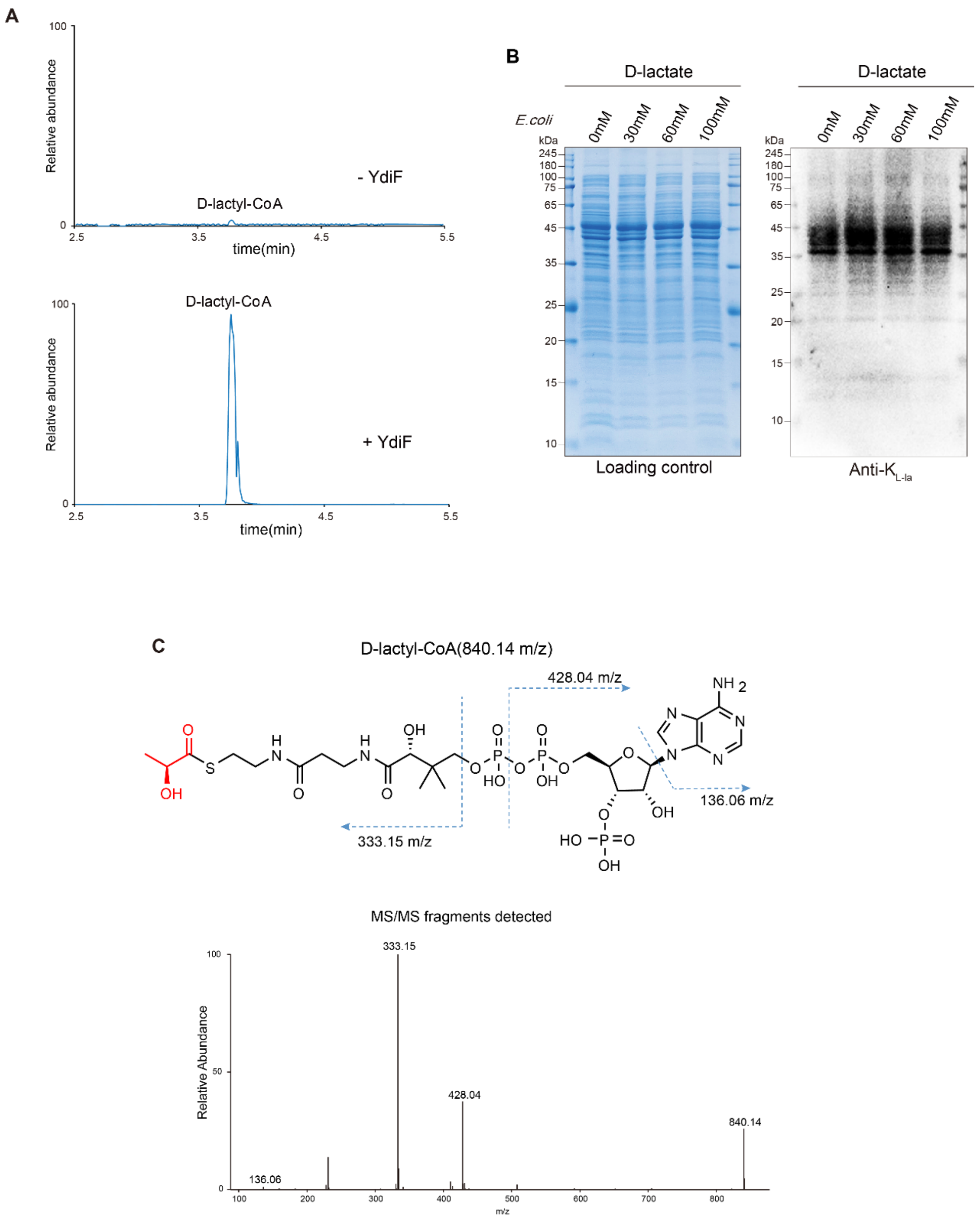

Supplementary Fig. 1. **YdiF catalyzes the generation of D-lactyl-CoA.** A, relative abundance of D-lactyl-CoA for YdiF reaction. B, *E. coli* wildtype strain was cultured in LB medium supplemented with 0, 30, 60, and 100 mM sodium D-lactate until the bacterial OD600 reached 0.4-0.5 at 37 °C. $K_{L\text{-}la}$ levels on whole-cell lysates were analyzed by immunoblotting. The loading control is identical to that in Figure 1D, as both parallel immunoblots were derived from the same biological samples. C, the chemical structure of D-lactyl-CoA with three MS/MS fragments. The MS/MS spectra

of D-lactyl-CoA analyzed by HPLC-MS/MS, including characteristic fragment ions and the precursor ion of 840.14 Da. All immunoblots had three biological repetitions.

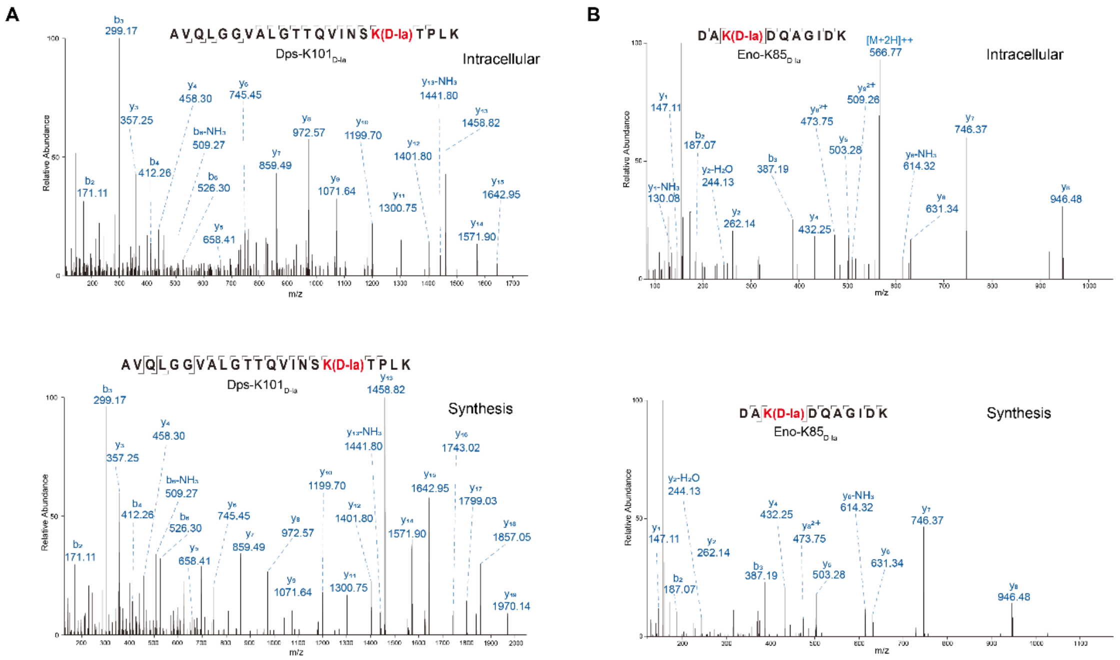

Supplementary Fig. 2. **The MS/MS spectra of identified K$_{D\text{-}la}$ peptides and its counterpart synthetic peptides.** A and B, the MS/MS spectra of the intracellularly modified peptides (AVQLGGVALGTTQVINSK(D-la)TPLK, Dps) and (DAK(D-la)DQAGIDK, Eno), compared to their synthetic counterparts.

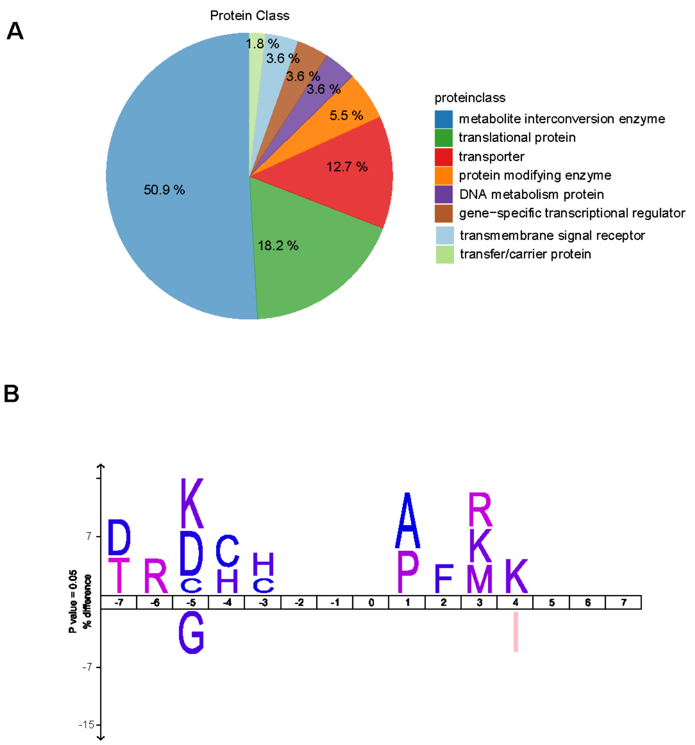

Supplementary Fig. 3. **Characterization of the $K_{D\text{-la}}$ proteome in *E. coli***. A, protein class analysis of $K_{D\text{-la}}$ proteins. B, sequence motif logo of $K_{D\text{-la}}$ sites generated by iceLogo software.

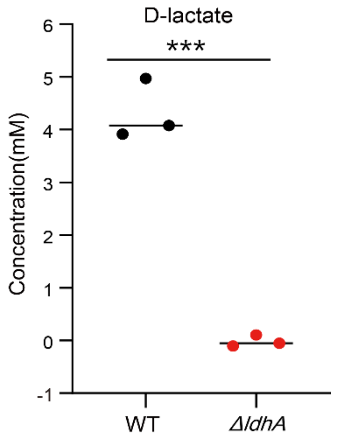

Supplementary Fig. 4. **LdhA regulates the generation of D-lactate under anaerobic.** The *E. coli* wildtype strain (WT) and Δ*ldhA* strain were cultured in LB medium under anaerobic conditions overnight at 37 °C. The supernatants were collected and analyzed using a D-lactate Assay Kit. Data represent mean ± S.D. from three independent biological replicates (n = 3).

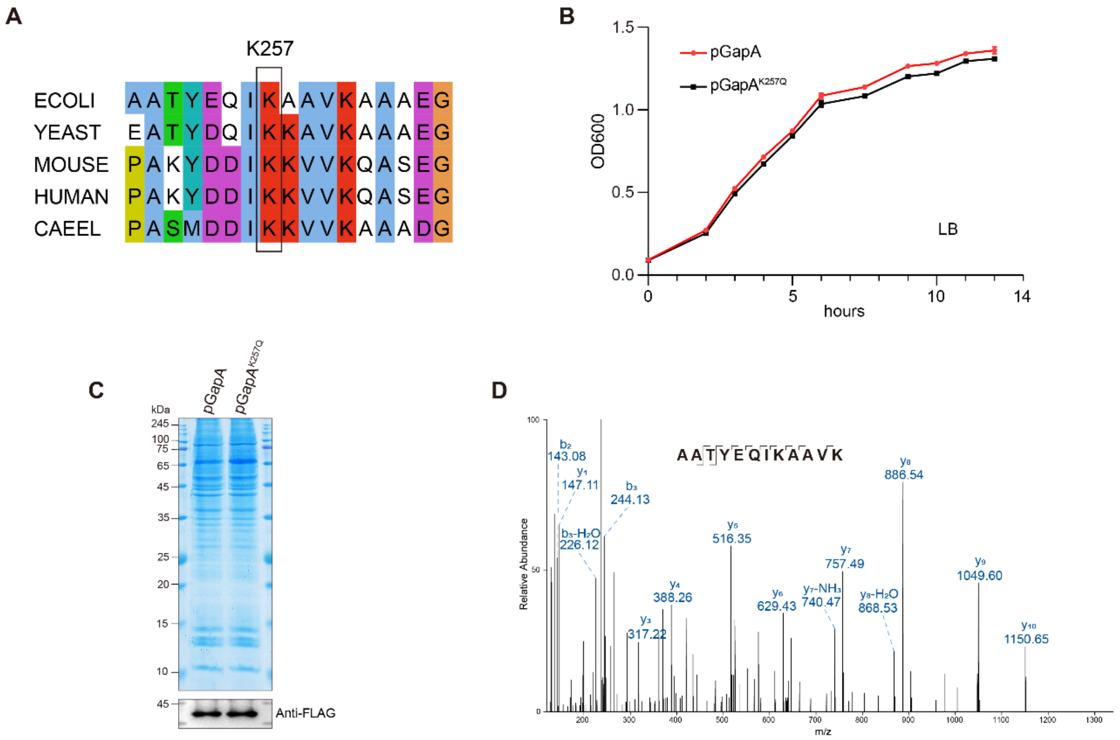

Supplementary Fig. 5. **CobB catalyzes the removal of lysine D-lactylation in vitro.** A, multiple sequence alignment of GapA homologs across five different species from bacteria to humans. B, growth curves of the pGapA and pGapA^{K257Q} strains inoculated into 15 mL tubes containing LB medium and cultured at 37 °C with shaking at 220 r.p.m. Growth was monitored by measuring OD600. C, immunoblotting confirmed equivalent expression of recombinant GapA and the K257Q mutant (GapA^{K257Q}) for GapA-overexpressing strains (pGapA and pGapA^{K257Q}) using an anti-FLAG antibody. D, the MS/MS spectra of the synthetic GapA K257$_{D\text{-}la}$ peptide (NLTGK(D-la)EADAALGR) after incubation with CobB, showing removal of the D-lactylation.

Supplementary Table 1. The data of D-lactate concentrations measured.

Related Fig. 1B and supplementary Fig. 4

	E. coli wildtype		Δ*ldhA*
D-lactate concentration (mM)	anaerobic	aerobic	anaerobic
	4.96834	-0.1416149	-0.0535821
	4.077868	-0.1025368	0.103552
	3.914784	0.1977112	-0.1023258

Supplementary Table 3. The data of YdiF with D-lactate reaction in vitro.

Related Fig.1E

	D-lactate+	
	D-lactyl-CoA intensity	Lg (D-lactyl-CoA intensity)
+YdiF	19700000	7.294466226
	91900000	7.963315511
	61000000	7.785329835
-YdiF	53300	4.726727209
	161000	5.206825876
	154000	5.187520721

Supplementary Table 6. The list of reagents, bacterial strains, and plasmids used in this paper.

REAGENT OR RESOURCE	SOURCE	IDENTIFIER
Antibodies		
Anti-D-Lactyl Lysine Rabbit mAb	PTM Biolabs	Cat#PTM-1429RM
Anti-D-Lactyl Lysine Rabbit pAb	PTM Biolabs	Cat#PTM-1429
Anti-D-Lactyl Lysine Antibody Conjugated Agarose Beads	PTM Biolabs	Cat# PTM-1434
Anti-L-Lactyl Lysine Rabbit mAb	PTM Biolabs	Cat#PTM-1401RM
Anti-FLAG mouse monoclonal antibody	Proteintech	Cat#66008-4-Ig
Bacterial Strains and Plasmids		
BL21 (λDE3) competent cells	TIANGEN	Cat# CB105
DH5α competent cells	TIANGEN	Cat# CB101
Escherichia coli MG1655	Tolo Biotech	N/A
cobB KO *Escherichia coli* MG1655	Tolo Biotech	N/A
E. coli Keio Parent Strain *BW25113*	Horizon Discovery	Cat# OEC5042
ydiF Keio *E. coli*	Horizon Discovery	Cat# OEC4988
ldhA Keio *E. coli*	Horizon Discovery	Cat# OEC4988
pET28a vector	Solarbio	Cat# P3110
pBR322 vector	MiaoLingBio	Cat# P0090
Synthetic Peptides		
NLTG-K(D-lactylation)-EADAALGR	Synpeptide Co Ltd	N/A
AATYEQI-K(D-lactylation)-AAVK	Synpeptide Co Ltd	N/A
AVQLGGVALGTTQVINS-K(D-	Synpeptide Co Ltd	N/A

lactylation)-TPLK		
DA-K(D-lactylation)-DQAGIDK	Synpeptide Co Ltd	N/A
PCR Primers		
pET28a (+)-F: GTGATGATGATGATGATGGCTGCT	This paper	N/A
pET28a (+)-R: GCACCACCACCACCACCACT	This paper	N/A
dps-pET28a-F: ACTTTAAGAAGGAGATATACCATGGGCAGCAGCCATCATCATCATCATCACATGAGTACCGCTAAATTAGT	This paper	N/A
dps-pET28a-R: CAGTGGTGGTGGTGGTGGTGCTTATTCGATGTTAGACTCGAT	This paper	N/A
gapA-pET28a-F: ACTTTAAGAAGGAGATATACCATGGGCAGCAGCCATCATCATCATCATCACATGACTATCAAAGTAGGTATC	This paper	N/A
gapA -pET28a-R: CAGTGGTGGTGGTGGTGGTGCTTATTTGGAGATGTGAGCGA	This paper	N/A
ldhA-pET28a-F: ACTTTAAGAAGGAGATATACCATGGGCAGCAGCCATCATCATCATCATCACAAACTCGCCGTTTATAGCAC	This paper	N/A

ldhA-pET28a-R: AGTGGTGGTGGTGGTGGTGCTT AAACCAGTTCGTTCGGGCAGG	This paper	N/A
dld-pET28a-F： ACTTTAAGAAGGAGATATACCAT GGGCAGCAGCCATCATCATCATC ATCACTCTTCCATGACAACAACT GA	This paper	N/A
dld-pET28a-R： AGTGGTGGTGGTGGTGGTGCTT ACTCCACTTCCTGCCAGTTTT	This paper	N/A
lldD-pET28a-F: ACTTTAAGAAGGAGATATACCAT GGGCAGCAGCCATCATCATCATC ATCACATTATTTCCGCAGCCAGC GA	This paper	N/A
lldD-pET28a-R: AGTGGTGGTGGTGGTGGTGCCT ATGCCGCATTCCCTTTCGCC	This paper	N/A
pBR322-F: CTCTAGAAGCGGCCGCGATC	This paper	N/A
pBR322-R: GGCAGCGACTACAAAGACCATG	This paper	N/A
gapA-pBR322-F: ATCGCGGCCGCTTCTAGAGGAA GACATGCCCCAGATGGGC	This paper	N/A
gapA-pBR322-R: GTCTTTGTAGTCGCTGCCTTTGG	This paper	N/A

AGATGTGAGCGATCAGGTC		
gapA-K257Q-pBR322-F: CAGGCTGCCGTTAAAGCTGCTG CTGAAGGCGAAATGAAAGGC	This paper	N/A
gapA-K257Q-pBR322-R: GCAGCTTTAACGGCAGCCTGGAT CTGCTCGTAAGTTGCAGC	This paper	N/A
ydiF-pBR322-F: TCGCGGCCGCTTCTAGAGACCTTCC GCAGTGCCAAAGA	This paper	N/A
ydiF-pBR322-R: GTCTTTGTAGTCGCTGCCATGAGCC GCTTCAGGCAGGA	This paper	N/A
Reagent		
HisPur Ni-NTA Resin	Thermo SCIENTIFIC	Cat# 88221
TransStart® FastPfu DNA Polymerase	TRANS	Cat# AP221-11
pEASY®-Basic Seamless Cloning and Assembly Kit	TRANS	Cat# CU201-02
D-lactate	Macklin	Cat#D742481-25g
D-Lactate Colorimetric Assay Kit	Elabscience	Cat# E-BC-K002-M
RIPA Lysis Buffer	Beyotime Biotechnology	Cat# P0013B
Deacetylase Inhibitor Cocktail	Beyotime Biotechnology	Cat# P1113

Glyceraldehyde-3-phosphate Dehydrogenase Activity Assay Kit	Solarbio	Cat#BC2210
Protein A+G Agarose (Fast Flow, for IP)	Beyotime Biotechnology	Cat# P2055
